# Supplementary figures and images for: CLEC5A and TLR2 are critical in SARS-CoV-2-induced NET formation and lung inflammation
Source: J Biomed Sci. 2022 Jul 11;29:52. doi: 10.1186/s12929-022-00832-z (PMC9277873; doi:10.1186/s12929-022-00832-z)

**Supplementary figure 1**

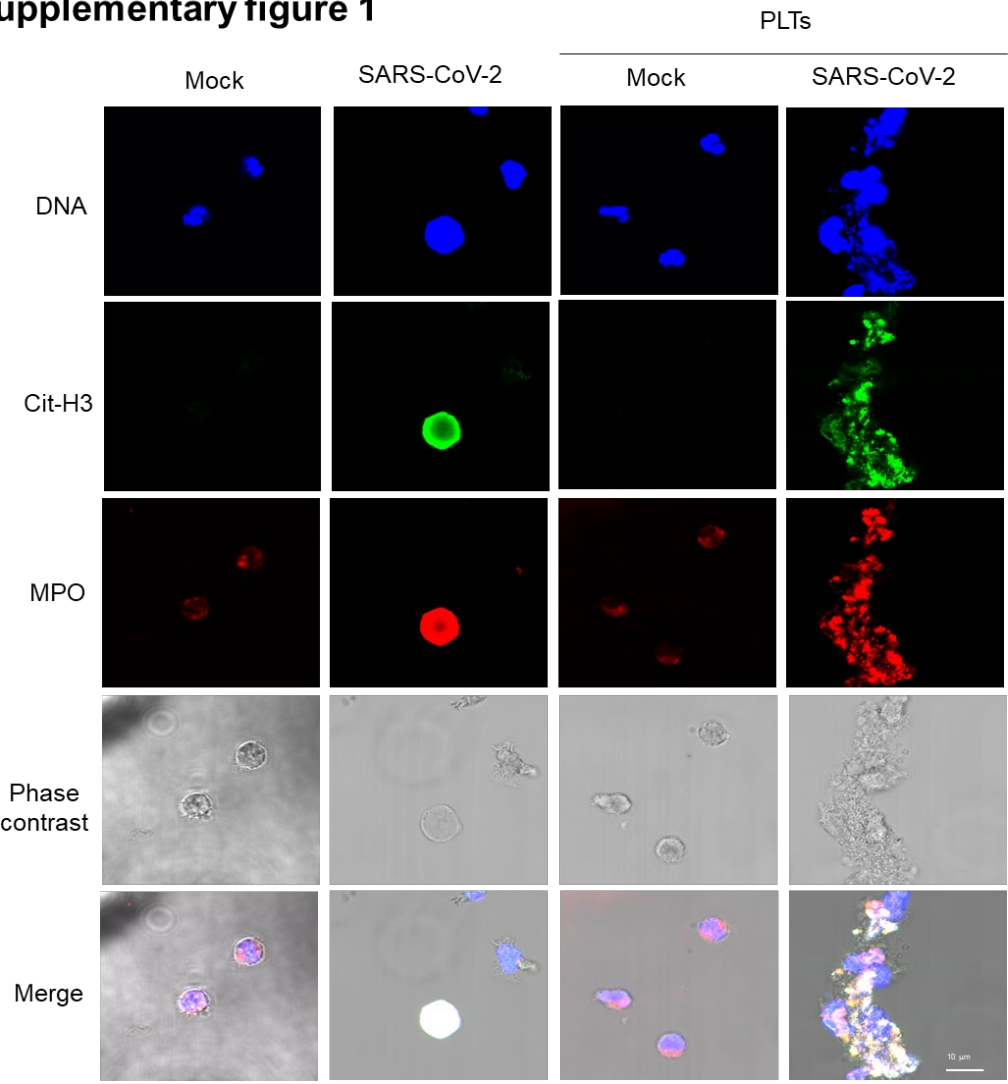

# Supplementary figure 2

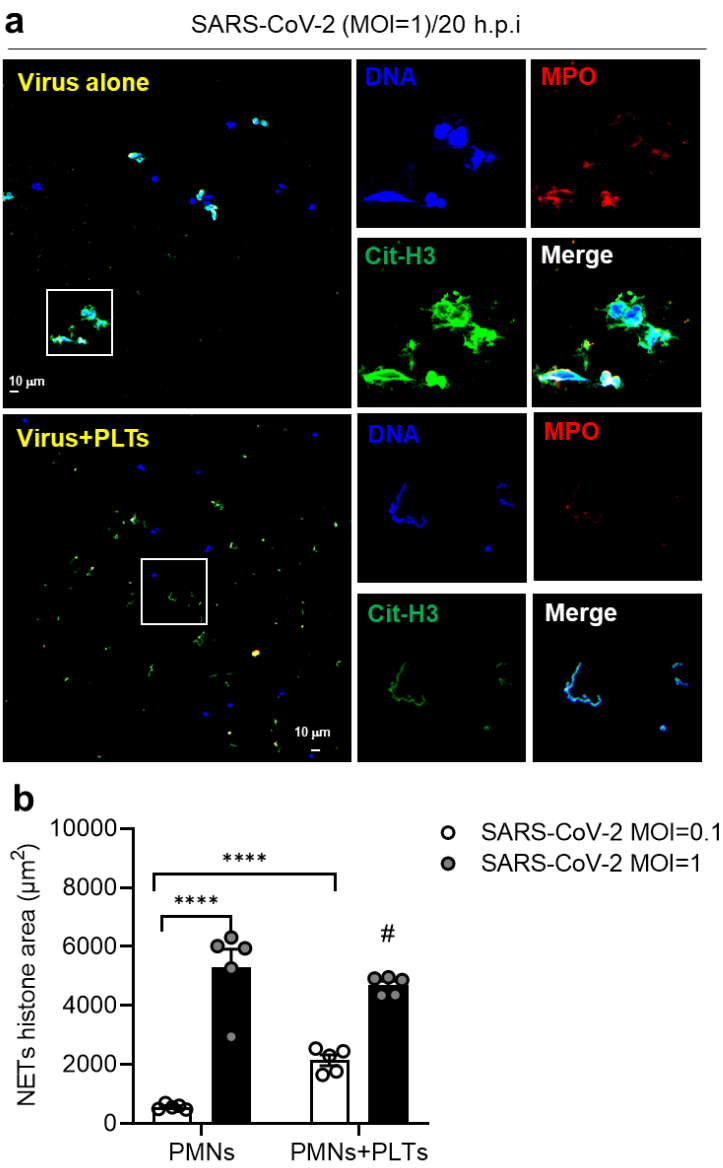

Supplementary figure 3

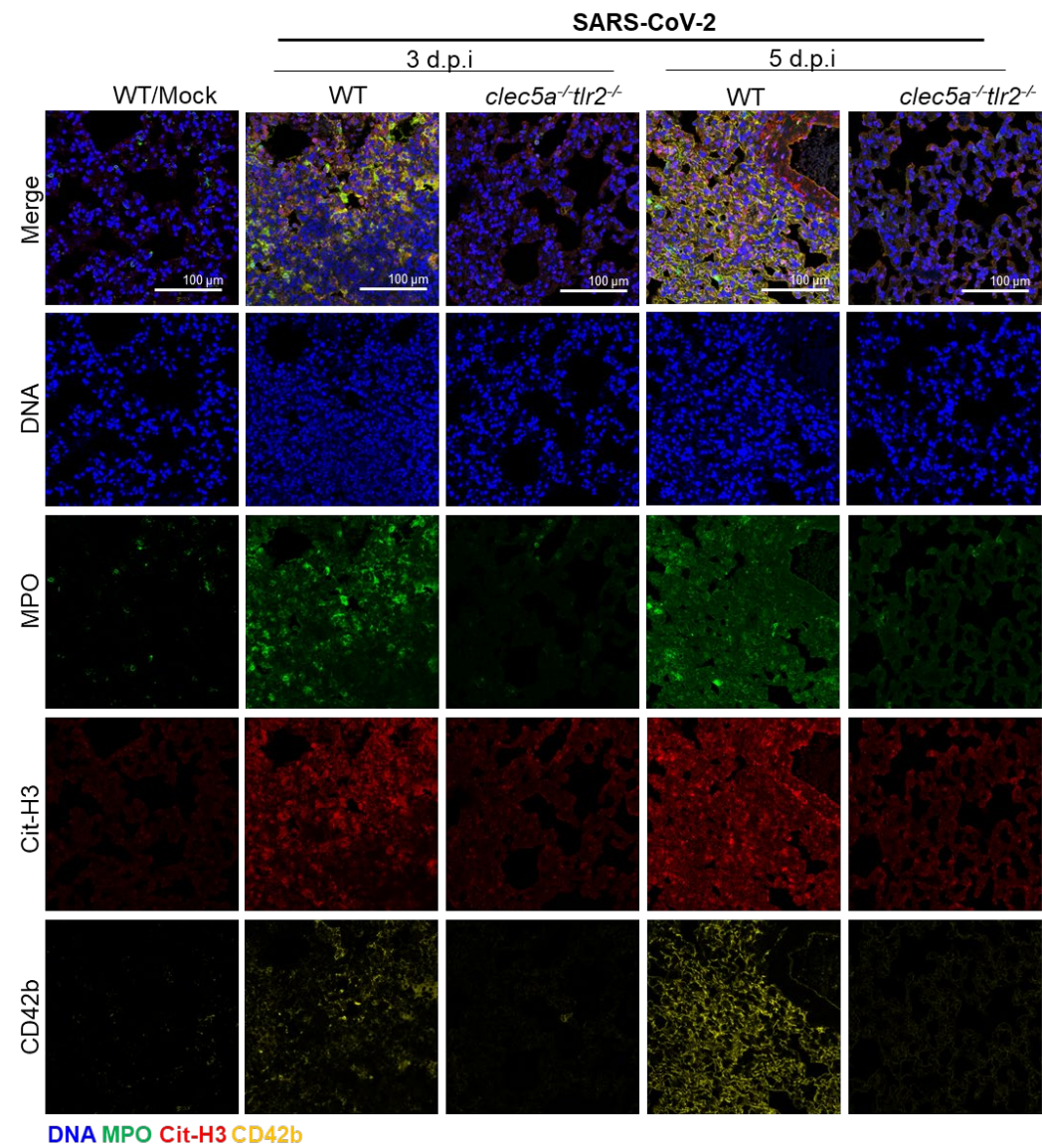

Supplement: Supplementary file 1 — Additional file 1: Figure S1. Platelet enhances SARS-CoV-2-induced NET formation. Human neutrophils (4 × 10^5^/ml) were incubated with SARS-CoV-2 (MOI = 1) in the presence or absence of autologous platelets (4 × 10^6^/ml) for 5 h at 37 °C. The NET structure was visualized by staining with DNA (blue), Cit-H3 (green), and MPO (red) then captured by confocal microscopy under 2000 × magnification. The colocalization of DNA, Cit-H3, and MPO was color in white. The scale bar is 10 μm. Figure S2. Platelet promotes severe NETs under SARS-CoV-2 stimulation after 20 h incubation. (a&b) Neutrophils (4 × 10^5^/ml) from healthy volunteers were stimulated with SARS-CoV-2 (MOI = 0.1 or 1) with or without autologous platelets (4 × 10^6^/ml) for 20 h at 37 ﾟC. Samples were fixed and stained with DNA (blue), Cit-H3 (green), and MPO (red). The image was captured by confocal microscopy under 400 × magnification (a). The scale bar is 10 μm. The level of NET was calculated using the image of Cit-H3 from 5 healthy donors (b). #: NET structure was detached from platelets and floated in culture supernatant. Data was presented as mean of area (μm2) ± SEM. **** p < 0.0001 (Student’s t-test). Figure S3. SARS-CoV-2-induced NETs and immunothrombosis are CLEC5A/TLR2-dependent. WT and clec5a−/−tlr2−/− mice were inoculated with AAV-ACE2 at day 14 day before SARS-CoV-2 challenge, and lung samples were collected at day 3 and day 5 post-infection. Tissue sections were stained with Hoechst 33342 for DNA (blue), anti-MPO antibody (green), anti-Cit-H3 antibody (red), and anti-CD42b antibody (yellow) to visualize the structure of NETs and immunothrombosis. Scale bar is 100 μm. [file 12929_2022_832_MOESM1_ESM.pdf]
