# Supplementary material for: CLEC5A and TLR2 are critical in SARS-CoV-2-induced NET formation and lung inflammation
Source: J Biomed Sci. 2022 Jul 11;29:52. doi: 10.1186/s12929-022-00832-z (PMC9277873; doi:10.1186/s12929-022-00832-z)
Supplement: Supplementary file 2 — Additional file 2: Table S1. Sequences of qPCR primers. [file 12929_2022_832_MOESM2_ESM.pdf]

Supplementary table 1. Sequences of qPCR primers

Target	Forward sequence (5’ to 3’)	Reverse sequence (5’ to 3’)
mouse *tnf-α*	GCCTCTTCTCATTCCTGCTTG	CTGATGAGAGGGAGGCCATT
mouse *il-6*	GAGGATACCACTCCCAACAGACC	AAGTGCATCATCGTTGTTCATAC
mouse *ip-10*	CCAAGTGCTGCCGTCATTTTC	GGCTCGCAGGGATGATTTCAA
mouse *cxcl1*	CAATGAGCTGCGCTGTCAGTG	CTTGGGGACACCTTTTAGCATC
mouse *cxcl2*	CCAAGGGTTGACTTCAAGAAC	AGCGAGGCACATCAGGTACG
mouse *cxcl5*	CCGCTGGCATTTCTGTTGCTGT	CAGGGATCACCTCCAAATTAGCG
mouse *ccl2*	TTAAAAACCTGGATCGGAACCAA	GCATTAGCTTCAGATTTACGGGT
mouse *ccl5*	GCTGCTTTGCCTACCTCTCC	TCGATGACAAACACGACTGC
mouse *ifn-γ*	CATGGCTGTTTCTGGCTGTTACTG	GTTGCTGATGGCCTGATTGTCTTT
mouse *gapdh*	GGAGAAACCTGCCAAGTATG	TGGGAGTTGCTGTTGAAG
